# Genome Sequences of the Listeria ivanovii subsp. ivanovii Type Strain and Two Listeria ivanovii subsp. londoniensis Strains

**DOI:** 10.1128/genomeA.01440-14

**Published:** 2015-01-22

**Authors:** Mario Hupfeld, Derrick E. Fouts, Martin J. Loessner, Jochen Klumpp

**Affiliations:** a Institute of Food, Nutrition and Health, ETH Zürich, Zürich, Switzerland; b J. Craig Venter Institute (JCVI), Rockville, Maryland, USA

## Abstract

We present the complete genomes of Listeria ivanovii subsp. ivanovii WSLC 3010 (ATCC 19119^T^), Listeria ivanovii subsp. londoniensis WSLC 30151 (SLCC 8854), and Listeria ivanovii subsp. londoniensis WSLC 30167 (SLCC 6032), representing the type strain of the species and two strains of the same serovar but different properties, respectively.

## GENOME ANNOUNCEMENT

Listeria are Gram-positive rod-shaped *Firmicutes* bacteria, currently split into nine species. L. ivanovii is an animal pathogen and primarily infects ruminants (1). We report the complete genome sequence of three L. ivanovii strains. One of them is the type strain of L. ivanovii subsp. ivanovii, which is very sensitive to infection by bacteriophages. In contrast, members of the subspecies londoniensis, such as WSLC 30151 and 30167, generally seem resistant to phage infection, except for the large virulent A511-like viruses.

L. ivanovii WSLC 3010 is a widely used serovar 5 isolate that is quite similar to the recently sequenced WSLC 3009 strain (2). While strains WSLC 30151 and WSLC 30167 also belong to the serovar 5 group, they have been designated L. ivanovii subsp. londoniensis, based on their inability to utilize certain sugars, such as ribose, and the degradation of *N*-acetyl-β-d-mannosamine (3).

Strains were grown at 30°C in BHI medium under aerobic conditions. Genomic DNA was prepared using the Sigma Genomic DNA Kit. DNA was subjected to single-molecule real-time sequencing on a Pacific Biosciences RS2 device (10-kb insert library, P4/C2 chemistry). Sequencing resulted in 135,255 reads with a 6,358-kb average length for WSLC 3010; 106,607 reads with a 6,780-bp average length for WSLC 30151; and 141,048 reads with a 6,244-bp average length for WSLC 30167. Genomes were assembled *de novo*, using SMRTAnalysis version 2.1.1 and the HGAP3 algorithm, into single contigs of 2,919,538 bp (WSLC 3010), 3,045,017 bp (WSLC 30151), and 2,993,492 bp (WSLC 30167), with 207-, 184-, and 229-fold average coverages. Genomes were annotated by the NCBI Prokaryotic Genomes Automatic Annotation Pipeline.

The WSLC 3010 genome contains 2,876 genes, 107 pseudogenes, and 67 tRNAs; the WSLC 30151 genome encodes 3,015 genes, 104 pseudogenes, and 67 tRNAs; and the WSLC 30167 genome features 2,930 genes, 90 pseudogenes, and also 67 tRNAs. The CRISPRFinder algorithm (4) was used to identify putative CRISPR loci within these strains. WSLC 3010 harbors three CRISPR regions, which are identical to those reported for WSLC 3009 (2). Two and three CRISPR regions were identified in WSLC 30151 and WSLC 30167, respectively. However, these CRISPR regions are significantly larger than those of related strains. Homologies of CRISPR spacers to different Listeria phages (e.g. B025, B054, PSA, and A500) were found in all three strains, and play a putative role in inactivation of invading phage DNA (5).

*Phage_Finder* (6) was used to screen the genomes for the presence of intact and cryptic phages. As previously reported for L. ivanovii WSLC 3009 (2), both L. ivanovii subsp. londoniensis WSLC 30167 and L. ivanovii subsp. ivanovii WSLC 3010 do not seem to harbor prophage sequences. One putative prophage region could be identified in L. ivanovii subsp. londoniensis WSLC 30151 at position 756388 to 792505 which is apparently inserted into a tRNA_Ser_ sequence. The prophage region features a mosaic structure, with homology to prophages of L. innocua Clip11262 (prophage Φ11262.6 [7]), L. welshimeri, and L. monocytogenes. Only strain WSLC 30151 featured a predicted monocin locus (8) of 15,297 bp, located at position 198267 to 213563.

### Nucleotide sequence accession numbers.

The complete genome sequences have been deposited in GenBank under the accession numbers CP009575, CP009576, and CP009577.
